# The Second Decade of Advanced Science – Expanding into New Areas

**DOI:** 10.1002/advs.202415954

**Published:** 2025-01-09

At the MRS Fall meeting in Boston, ten years ago, we celebrated a new addition to the Advanced Portfolio: ***Advanced Science***. Although it started small with just a single issue featuring 5 papers published in December 2014, the launch represented a milestone in the history of the Advanced Portfolio, as it was the first gold open‐access title in the group, at a time when the concept of open access was still relatively new.

**Figure 1:**
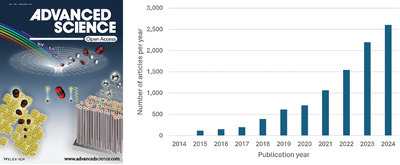
The first cover published in the first issue of Advanced Science in December 2014, Volume 1, Issue 1 (left); number of articles published in Advanced Science from 2014 to 2024 [Data from Web of Science, November 2024] (right).

The journal got indexed on Web of Science in 2015, and received its first impact factor in 2016, which spurred the rapid development of the journal. To date, ***Advanced Science*** has published nearly 10,000 article (**Figure** **1**), and it has established itself as a leading journal through the dedication and teamwork of the Advanced editorial team.

Today, ***Advanced Science*** is recognized globally for its outstanding contribution to scientific publishing. It is a platform for sharing discoveries, and inspiring readers, and serves as a beacon for those who pursue progress and aim to share it widely. ***Advanced Science*** welcomes contributions from across physical sciences, engineering, biology, environmental science, social sciences, as well as health and life sciences.

To mark this memorable 10th anniversary of ***Advanced Science***, we have been honored and proud to celebrate this important milestone with our authors, our community, and our editors.

In February, we published our Anniversary Special Issue with 29 reviews and perspectives exclusively written by our editorial board members on the most prominent topics in materials science; from chemoproteomics and neuroimmune interaction to liquid‐crystalline polymer membranes, MXenes for aqueous zinc batteries, and many more.

In addition, a selection of handpicked articles, authored by experts across the world and curated by our editorial team was collected into a “10th anniversary” virtual collection to showcase more breakthroughs published in the journal during the year.

**Figure 2:**
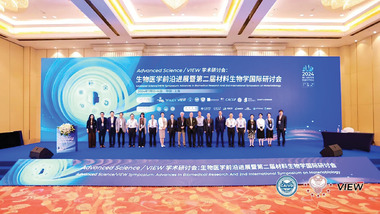
Photo of the event hosted in Shanghai, China, July 12th‐14th, 2024. From left to right: Prof. Rongrong Zhu, Dr. Guangchen Xu, Prof. Liming Zhao, Prof. Haibo Song, Dr. Luzhuang Guo, Dr. Kirsten Severing, Mr. Qing Pan, Ms. Rong Chen, Prof. Changsheng Liu, Prof. Buddy D. Ratner, Prof. Nicholas A. Peppas, Prof. Kam W. Leong, Prof. Zhinan Chen, Mr. Philip Kisray, Prof. Songguo Zheng, Prof. Hualiang Wang, Prof. Ping Zhu, Ms. Yuanqian Zeng, Ms. Yanting Lu, Prof. Kun Qian.

Throughout the year, our editorial team organized three major events, in China, Germany, and the USA, to commemorate the anniversary. The first in‐person event took place in Shanghai in July 2024, where over 60 globally renowned scientists and scholars from around the world were invited to present their work. They explored the latest advancements and future trends in the field of biomaterials science with more than 340 attendees (**Figure** **2**).

**Figure 3:**
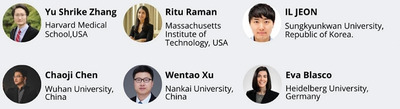
The winners of the Advanced Science Young Innovator Award 2024.

During the symposium, the Advanced Science Young Innovator Award was presented to six young scientists for their exceptional contributions to the fields of materials science, physics and chemistry, medicine and life sciences, and engineering (**Figure** **3**).

**Figure 4:**
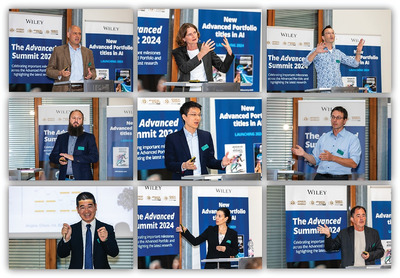
Photos from the Advanced Summit hosted in Weinheim, Germany on September 11th, 2024. From left to right; 1st^st^ row: Prof. Dr. Paolo Samorí, Prof. Dr. Tanja Weil, Prof. Dr. Stefan Hecht; 2nd^nd^ row: Prof. Dr. Martin Kaltenbrunner, Prof. Zijian Zheng, Prof. Dr. Pavel Levkin; 3rd^rd^ row: Prof. Nanfeng Zheng, Prof. Dr. Eva Blasco, Prof. Dr. Martin Heilmaier. Prof. Zhiyong Tang attended the event remotely.

In September 2024, we hosted the “Advanced Summit” at our German headquarters in Weinheim. We invited 10 high‐profile researchers in the fields of polymer science, optoelectronics, engineering, and technology, to showcase their most recent findings (**Figure** 4). The presentations were broadcast live to more than 1600 online participants. The lectures can be rewatched through a recording available by following this link: https://advanced.onlinelibrary.wiley.com/hub/summit.

As our 10th anniversary publication year came to a close, our editors made a meaningful return to our roots by attending the MRS conference in Boston in early December 2024 for their third big in‐person event. This connection with the MRS community fostered once again impactful collaborations and strengthened relationships, driving ***Advanced Science*** to continue showcasing cutting‐edge research from talented scientists.

**Figure 5:**
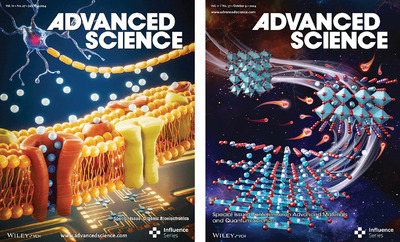
Front cover of the “Organic Bioelectronics” special issue, Volume 11, Issue 27 (left); front cover of the “Advanced Materials and Quantum Science” special issue, Volume 11, Issue 37 (right).

In addition to the 10th Anniversary Special Issue mentioned above, two outstanding topical collections were published in the journal in 2024. The first, focused on Organic Bioelectronics, explores both the fundamentals and applications of organic electronics materials in biology and medicine. Guest‐edited by Mohammad Reza Abidian (University of Houston, USA), the collection includes contributions from all over the world dedicated to this emerging field. The second, focused on Advanced Materials and Quantum Science, was guest‐edited by Shulei Chou and colleagues at Wenzhou University in China. Published in July 2024, this collection explores revolutionary advances covering a range of applications from computer science to solar cells, catalysis, microfluidics, biomedical technologies, sensors, etc (**Figure** **5**).

As always, we evaluated the topics that generated the greatest interest from our readership by examining metrics such as citations and Altmetric scores.

The top‐cited and the most influential papers in 2024, our 10th anniversary year, reflect the diverse research areas and broad scope of ***Advanced Science*** (**Table** **1**).

**Table 1:** Top 10 most‐cited Advanced Science articles in 2024, published in 2022–2023 (data extracted on November 29th, 2024).

	Title	Corresponding Author(s), Affiliation(s)	Published in	Citations in 2024 (Web of Science	Total Citations (Web of Science)
1	Dielectric Loss Mechanism in Electromagnetic Wave Absorbing Materials	Hongjing Wu, Northwestern Polytechnical University, Xi'an, China	February 2022	291	674
2	Electrolyte Solvation Structure Design for Sodium Ion Batteries	Jun Ming and Husam N. Alshareef, Chinese Academy of Sciences, Changchun, China and KAUST, Saudi Arabia	June 2022	176	274
3	A Highly Sensitive CRISPR‐Empowered Surface Plasmon Resonance Sensor for Diagnosis of Inherited Diseases with Femtomolar‐Level Real‐Time Quantification	Zhongjian Xie and Han Zhang, Shenzhen International Institute for Biomedical Research, Shenzhen and Shenzhen University, China	March 2022	165	184
4	Immunogenic Cell Death Activates the Tumor Immune Microenvironment to Boost the Immunotherapy Efficiency	Xuelei Ma and Kui Luo, Sichuan University, Chengdu, China and Chinese Academy of Medical Sciences, Chengdu, China	June 2022	150	264
5	Engineering Robust Ag‐Decorated Polydopamine Nano‐Photothermal Platforms to Combat Bacterial Infection and Prompt Wound Healing	Wei Dong, Fangfu Ye, and Jianliang Shen, Nanjing University of Science and Technology, China, and University of Chinese Academy of Sciences, Wenzhou, China	February 2022	139	311
6	Ionic Conduction in Polymer‐Based Solid Electrolytes	Xin Guo, Huazhong University of Science and Technology, China	January 2023	135	181
7	Exosome Processing and Characterization Approaches for Research and Technology Development	James J. Lai, University of Washington, Seattle, WA, 98195 USA	March 2022	117	187
8	Rational Design of Better Hydrogen Evolution Electrocatalysts for Water Splitting: A Review	Zhen‐Feng Huang and Ji‐Jun Zou, Tianjin University, China	April 2022	115	232
9	Exosomes‐Loaded Electroconductive Hydrogel Synergistically Promotes Tissue Repair after Spinal Cord Injury via Immunoregulation and Enhancement of Myelinated Axon Growth	Yongjian Sun, Lei Zhou, and Chengyun Ning, Southern Medical University, Guangzhou, China and South China University of Technology, Guangzhou, China	March 2022	113	209
10	Recent Advances and Strategies toward Polysulfides Shuttle Inhibition for High‐Performance Li‐S Batteries	Qingshui Xie and Dong‐Liang Peng, Xiamen University, China	March 2022	98	235

**Figure 6:**
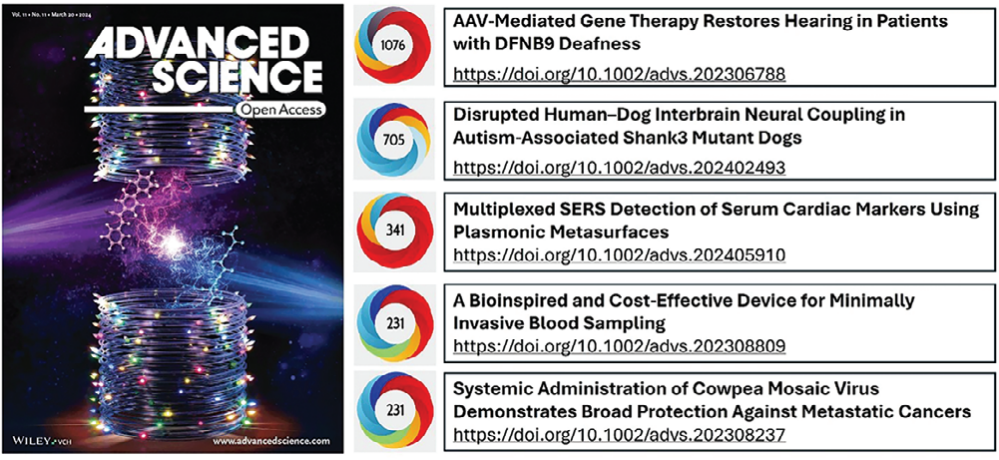
Articles published in Advanced Science in 2024 with the highest Altmetric score [Data from Dimensions, November 2024].

**Figure** **6**. shows articles published in 2024 that registered the highest Almetric score. The article entitled “AAV‐mediated gene therapy restores hearing in patients with DFNB9 deafness” attracted the highest interest and engagement with our readership and demonstrates restored or significantly improved hearing in patients after cochlear administration of dual AAV‐OTOF vectors.

**Figure 7:**
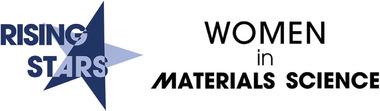
Logos of the Rising Stars series (left) and Women in Materials Science virtual collection (right).

Championing the diverse, international community of our journal and the desire to inspire future generations of scientists, ***Advanced Science*** renewed its efforts in the Rising Stars Series and the Women in Materials Science Virtual Issue with many newly selected articles and authors (**Figure** **7**).

**Figure 8:**
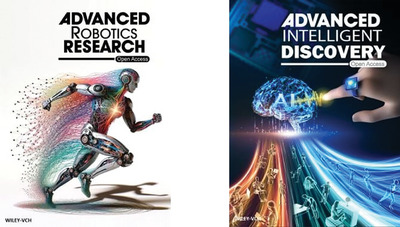
Cover of Advanced Robotics Research (left) and Advanced Intelligent Discovery (right).

Much like when *Advanced Science* was introduced in Boston a decade ago, the MRS Fall 2024 conference served as the platform for introducing two new gold open‐access titles of the Advanced Portfolio, Advanced Robotics Research and Advanced Intelligent Discovery (**Figure** **8**). These new titles build on the success and reputation of Advanced Intelligent Systems in the areas of Robotics and Artificial Intelligence. As evidenced by the 2024 Nobel prizes in Physics and Chemistry being awarded to work that heavily featured artificial intelligence, the launch of these new titles is extremely timely and provides another avenue for our authors to publish their work in the Advanced Portfolio and push the limits of our knowledge.

As we celebrated the journal's 10th anniversary, we built on a decade of success by expanding the focus areas of *Advanced Science*, paving the way for even greater contributions to the scientific community. While maintaining a strong focus on materials science, technology, engineering, and physics we gaze more toward Life and Health sciences by including 7 new research areas: *Genetics & Genomics* focus is foundational to modern scientific advances, covering genetic engineering to population genetics; *Cell & Molecular Biology* explores life at cellular and molecular levels, promising novel therapeutic insights; *Neuroscience & Neurology* delves into the brain and nervous system, aiming to develop innovative treatments; *Oncology & Cancer Biology* advances cancer research, striving to contribute to the global fight against cancer; *Geroscience & Aging Biology* explores aging processes to improve health span and lifespan; *Food & Agriculture* tackles global challenges in sustainable food production and security; *Plant Science* addresses environmental and agricultural challenges, driving sustainable practices.

To support these expanded areas, we have welcomed additional professional editors with expertise in their fields, each dedicated to upholding the highest standards of quality and fairness: Lei Lei (Deputy Editor for Plant Sciences), Yuming Hu (Deputy Editor for Genetics & Genomics), Paulina Strzyz (Deputy Editor for Cell & Molecular Biology), Andrew Jobbins (Deputy Editor for Neuroscience & Neurology), Ada Wong (Deputy Editor for Oncology & Cancer Research), Taolan Zhao (Deputy Editor for Food & Agriculture), and Monty Montano (Editor, Geroscience & Aging Biology).

With a decade of success behind us, ***Advanced Science*** has become a trusted source of high‐quality research, and our expanded editorial team and newly broadened focus areas aim to better serve the global research community in the years ahead.

As we embark on a new decade, we look forward to the groundbreaking discoveries that will shape the future of science, and we wish to thank you, all our Authors and Readers, for trusting us and for your continued support as we enter the next phase of our journey.

On behalf of the whole team,

*Kirsten Severing, Valentina Lombardo, Lei Lei, Alanna Gannon, Richard Murray, Shaoying Cui*

